# A novel intervention to reduce noninfectious and infectious complications associated with indwelling urethral catheters in hospitalized older patients: a quasi-experimental study

**DOI:** 10.1186/s12877-022-03113-4

**Published:** 2022-05-16

**Authors:** Fang-Wen Hu, Chun-Yin Yeh, Chi-Chang Huang, Hsiu-Chi Cheng, Cheng-Han Lin, Chia-Ming Chang

**Affiliations:** 1 Department of Nursing, National Cheng Kung University Hospital, College of Medicine, National Cheng Kung University, No. 138, Sheng-Li Road, Tainan City, 70403 Taiwan; 2 Department of Computer Science and Information Engineering, National Cheng Kung University, No. 1, University Road, Tainan City, 70101 Taiwan; 3 Department of Geriatrics and Gerontology, National Cheng Kung University Hospital, College of Medicine, National Cheng Kung University, No. 138, Sheng-Li Road, Tainan City, 70403 Taiwan; 4 Department of Internal Medicine, and Institute of Clinical Medicine and Molecular Medicine, National Cheng Kung University Hospital, College of Medicine, National Cheng Kung University, No. 138, Sheng-Li Road, Tainan City, 70403 Taiwan; 5 Department of Internal Medicine, Tainan Hospital, Ministry of Health and Welfare, 125 Jhongshan Rd, West Central Dist, 70043 Tainan City, Taiwan; 6 Division of Geriatrics and Gerontology, Department of Internal Medicine, College of Medicine, National Cheng Kung University Hospital, National Cheng Kung University, No. 138, Sheng-Li Road, Tainan City, 70403 Taiwan

**Keywords:** Complications, Hospitalized, Intervention, Older adults, Urinary catheters

## Abstract

**Background:**

Indwelling urethral catheters are widely used in clinical settings. Catheter-associated urinary tract infection has been recognized as a common adverse event in older patients. However, noninfectious complications are almost 5 times as common as infectious complications, and insufficient attention has been given to noninfectious complications. Given this importance, a novel intervention related to removing unnecessary catheters in a timely manner to promote, after removal, the recovery of self-voiding function is herein developed to reduce infectious and noninfectious complications associated with indwelling urethral catheters in hospitalized older patients.

**Methods:**

A quasi-experimental study design was adopted. Patients aged 65 and older who had a urinary catheter placed within 24 h of hospital admission were included. All patients were allocated into either an intervention group, in which the novel intervention developed in the study was implemented, or a control group, who received care as usual. The outcomes of this study were to evaluate whether the novel intervention reduced the incidence of the following: catheter-associated urinary tract infections, catheter-associated noninfectious complications, decline in activities of daily living, and new nursing home admissions.

**Results:**

Of 106 hospitalized older patients who consented to participate, 92 completed follow-up until discharge, including 49 in the control group and 43 in the intervention group. The patients in the intervention group were significantly older than those in the control group [83.72 ± 9.18 vs. 80.26 ± 7.66, *p* = 0.038], and no differences were found between the groups in other demographics or present health conditions. Multivariable logistic regression analysis showed that the control group was more likely to develop noninfectious complications [adjusted odds ratio: 3.01, 95% confidence interval: 1.32–6.81] and a decline in ADLs [adjusted odds ratio: 11.20, 95% confidence interval: 3.68–34.00].

**Conclusions:**

A novel intervention can be effective as a means of reducing noninfectious complications associated with indwelling urethral catheters in hospitalized older patients. This approach will help to standardize urethral catheter care, and it highlights the fact that health care professionals can play a crucial role in preventing harm from urethral catheters.

**Supplementary Information:**

The online version contains supplementary material available at 10.1186/s12877-022-03113-4.

## Background

Indwelling urethral catheters are widely used in clinical practice, and approximately 19.8–61.1% of patients are catheterized during their hospital stay [1]. Catheter-associated urinary tract infection (CAUTIs) has been recognized as a common adverse event that may lead to bacteraemia and death, especially in acutely ill elderly patients [2]. However, Hollingsworth et al. (2013) reported in their meta-analysis of 37 studies that noninfectious complications were as common as CAUTIs [3]. Saint et al. (2018) also conducted a multicentre cohort study of 2,076 patients with indwelling urethral catheters, in which noninfectious complications (55%) were 5 times as common as infectious complications (11%) [4]. Insufficient attention has been given to noninfectious complications such as pain or discomfort, bladder or kidney stones, paraphimosis, meatal erosion, and gross haematuria [5]. In addition, indwelling urethral catheters have been equated to a one-point restraint, which may cause pressure injuries and a decline in activities of daily living (ADLs), which may further increase the incidence of new nursing home admissions [6].

Eliminating unnecessary urethral catheter use is certainly the most important goal in preventing catheter-associated adverse outcomes. Currently, reminders for the removal of unnecessary catheters as soon as possible are well known to minimize the inappropriate use of urethral catheters [1, 7, 8]. However, (Reference removed for blind review) indicated that 20.6% of hospitalized older patients underwent urethral catheter reinsertion on the same day and that up to 49.5% of such catheter reinsertions were preventable [6]. Unfortunately, no strategy was given related to removing unnecessary catheters in a timely manner and promoting the recovery of self-voiding function after catheter removal in hospitalized older patients. In light of the high incidence of noninfectious complications, urethral catheter-associated noninfectious complications should be vital targets for future preventive efforts [4]. The purpose of this study was to develop a novel intervention that consists of strategies for reducing inappropriate catheter use and promoting recovery of self-voiding function. A further aim was to explore the effects of the intervention in terms of reducing infectious and noninfectious complications in hospitalized older patients.

## Methods

### Study design and participants

This study had a quasi-experimental design that compared a novel intervention to reduce complications associated with indwelling urethral catheters against usual care in hospitalized older patients. Participants were recruited from the adult wards of a tertiary-care medical centre in southern Taiwan. Patients aged 65 and older who had a urethral catheter inserted within 24 h of hospital admission were included. The exclusion criteria were as follows: 1) immediately requiring intensive care or 2) needing hospice care or surgery. Older patients admitted to adult wards from October 2017 through February 2018 comprised the control group, and those admitted from March through July 2018 made up the intervention group. In the intervention group, the novel intervention developed in this study was implemented and followed up by the research nurse. Care involving urethral catheters was performed as usual in the control group.

### Study intervention

We conducted an extensive literature review to organize and determine the contents of the novel intervention, which we divided into two parts. The first part aimed to ensure the appropriate use of urethral catheters [6, 9–12] and to remove unnecessary catheters in a timely manner. The second part of the intervention aimed to promote the recovery of self-voiding function after catheter removal, including the assessment of risk factors for urine retention and the implementation of strategies to promote self-voiding [13–15].

The drafted flow diagram of the novel intervention was revised through consultation with a multidisciplinary panel of five experts consisting of two geriatricians and one gerontological clinical nurse specialist, one urologist, and one physical medicine and rehabilitation physician. Two sequential rounds of anonymous questionnaires were conducted and the consensus on the flow diagram by the experts in round 2 was 98%.

**Fig. 1:**
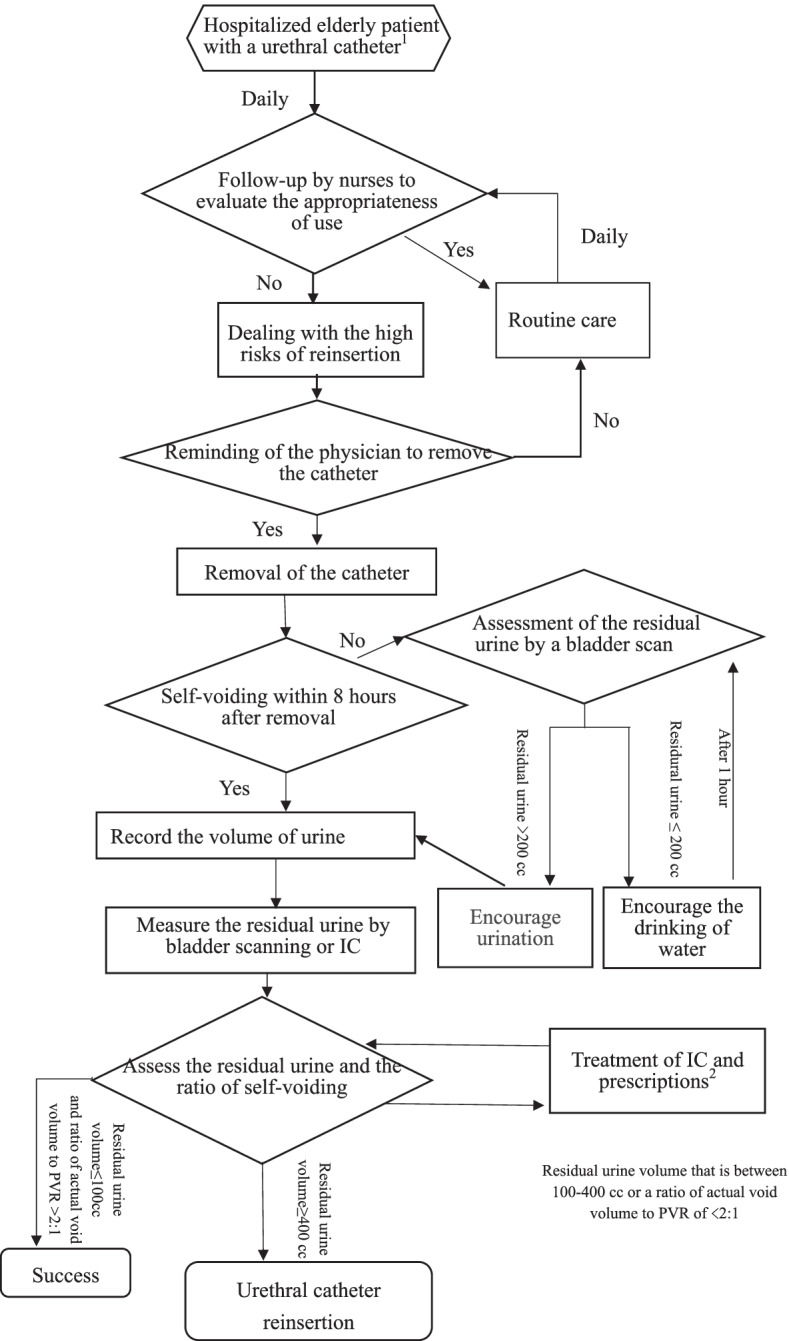
Flow diagram of a novel intervention. Note: 1. Record the urine volume and reason for urethral catheter insertion. 2. Apply intermittent catheterization (IC) Q12H when the first-time postvoid residual volume (PVR) is 100–200 ml; IC Q8H, when the PVR is 200–300 ml; and IC Q6H, when the PVR is > 300 ml. Successful removal of the catheter is defined as PVR ≤ 100 ml or a ratio of the actual void volume to the postvoid residual volume of > 2:1

Finally, face-to-face interviews were conducted to understand the first-line nurses’ considerations in implementing this intervention in clinical practice, and the content of the intervention was refined according to their thoughts and feelings at the beginning of the study. The final version of the flow diagram is shown in Fig. 1. Hospitalized older patients with urethral catheters will be followed up by nurses to evaluate the appropriateness of use every day. The indication for appropriate use of urethral catheters included medication instillation or bladder irrigation; dysuria due to bladder outlet obstruction without better solutions; urinary retention ≥ 400 cc without better solutions; close monitoring of urine output in critically ill patients; perioperative management; open sacral or perineal wounds with a need for urinary diversion in incontinent patients; and special needs, including the considerations of medical members and needs in the physiological, psychological, and social domains among hospitalized older patients. If a urethral catheter is no longer necessary, the clinician would need to address the high risks of reinsertion before attempting to remove it. The risk factors for urine retention include obstruction of the bladder, urinary tract infection, urinary tract trauma, drug induction, neuropathy, and external factors. If risk factors for urine retention are eliminated, the physician should be reminded to remove the catheter. After catheter removal, strategies to promote self-voiding include establishing a urination schedule, encouraging patients to regularly urinate every 2 to 4 h, monitoring water intake, selecting the Valsalva manoeuvre or Crede’s method, and creating a voiding diary. If older patients have self-voiding within 8 h after removal, the volume of urine needs to be recorded, and the residual urine needs to be measured by bladder scanning or intermittent catheterization to assess the residual urine and the ratio of self-voiding.

### Measurements and procedure

Ethical approval was obtained from the institutional review board of National Cheng Kung University Hospital (IRB NO. B-ER-106–152). The purpose and process of this study were explained to potential participants. Written informed consent was obtained from each participant, after which, baseline data collection was conducted by the researcher within 48 h of admission. During the baseline data collection, medical records were reviewed, and the patient was interviewed. The medical records provided demographic factors (age and sex) and present health conditions (chronic constipation history, genitourinary surgery history, Charlson Comorbidity Index (CCI), and body mass index). The CCI indicated the number and severity of comorbidities with scores ranging from 0 to 37, with higher scores indicating more severe comorbidities [16]. In addition, interviews were conducted regarding urinary incontinence, cognitive function, and depressive symptoms. Urinary incontinence was defined as the experience of wetting oneself within the previous 2 weeks by the patient’s self-report [17]. The main caregivers were asked if the older patients were incompetent in communication. The Short Portable Mental Status Questionnaire (SPMSQ) referred to the cognitive function of older patients, and cognitive impairment was defined as 2 or more errors after adjusting for education level on the SPMSQ [18]. The SPMSQ was automatically coded as cognitive impairment in patients without competence to respond. The Geriatric Depression Scale Short-Form (GDS-SF) identified depressive symptoms in older patients with a total score of more than 8 [19]. The GDS-SF was not measured among patients whose communication was inadequate. Accordingly, observations were made by the researcher to assess ADLs. The Katz ADL score was measured using 6 items (impairment in bathing, dressing, visiting the toilet, getting up out of a chair, eating, the use of incontinence materials) with scores ranging from 0–12, and higher scores indicated more independence in ADLs [20].

After baseline data were collected, the older patients in the intervention group underwent a novel intervention and was followed by the research nurse (Fig. 1). Patients in the control group received usual care. Adverse outcomes were collected at discharge by the first researcher, which included the incidence of CAUTIs and catheter-related noninfectious complications, ADL decline, and new nursing home admissions. All outcomes except ADL decline were collected from medical records, including physician progress notes and nursing records. CAUTIs were operatively defined as the presence of a CAUTI diagnosis in medical records during admission. Catheter-related noninfectious complications were defined as the patient having one of the following diagnoses after catheterization: internal trauma (pain, discomfort, blood in the urine [incidentally noted upon catheter removal], and overt mechanical trauma), external trauma (gangrene of the penis, paraphimosis, and meatal erosion), fistula, urinary leakage or incontinence, and protective constraint from accidental catheter removal [3, 5]. ADL decline was assessed by the first researcher and was defined as a reduction in the Katz ADL scores from admission to discharge. New nursing home admission was defined as when a patient was discharged to a nursing home whereas he or she had not been residing in one before admission.

### Statistical analysis

The data were analysed using IBM SPSS Statistics 19 software. Patients’ basic information was displayed using the frequency, percentage, mean, standard deviation, and median and interquartile range. The differences between the intervention and control groups were examined using independent t tests and Wilcoxon rank sum tests for continuous variables and χ2 tests for categorical variables. Multivariable logistic regression was conducted to determine the effects of the intervention on adverse outcomes. All results were considered statistically significant at a *p* value < 0.05.

## Results

### Participant characteristics

A total of 487 older patients were approached for eligibility screening. Of the 128 patients who met the inclusion criteria, 106 consented to participate, and half of them became the intervention group. Withdrawal as a result of deterioration in medical status and admission to the intensive care unit (ICU) occurred in 11.3% of patients in the intervention group (6/53) and 3.7% of the control group (2/53); 7.5% of the intervention group (4/53) and 3.7% of the control group (2/53) were transferred to hospice care. No significant difference was found between those who remained and withdrew from this study in terms of demographic and health characteristics.

Demographic factors and present health conditions from the 106 patients at baseline are shown in Table 1. The mean age of the patients was 81.99 (SD = 8.59), and 53.8% (*n* = 57) were female. The only demographic variable that differed significantly between the groups was age (*p* = 0.038); the average age was 83.72 years (SD = 9.18) in the intervention group compared to 80.26 years (SD = 7.66) in the control group. No differences were found between the groups in present health conditions.

**Table 1:** Characteristics of the intervention and control groups

Variables	Overall (*n* = 106)	Intervention group (*n* = 53)	Control group (*n* = 53)	*p*
**Demographic factors**				
Age^a^	81.99 ± 8.59	83.72 ± 9.18	80.26 ± 7.66	0.038
Female^b^	57 (53.8%)	26 (49.1%)	31 (58.5%)	0.330
**Present health conditions**				
Chronic constipation history^b^	34 (32.1%)	17 (32.1%)	17 (32.1%)	1.000
Genitourinary surgery history^b^	27 (25.5%)	12 (22.6%)	15 (28.3%)	0.504
Urinary incontinence^b^	43 (40.6%)	22 (41.5%)	21 (39.6%)	0.843
Charlson comorbidity index^c^, median ± IQR	6 (4–7)	6 (4–7)	6 (3.5–7.5)	0.929
Body mass index^a^	22.52 ± 4.18	22.39 ± 4.16	22.65 ± 4.64	0.749
Cognitive impairment^b^	82 (77.4%)	42 (79.2%)	40 (75.5%)	0.643
Depressive symptoms^b^	36 (34.0%)	20 (54.1%)	16 (36.4%)	0.110
Katz ADL score (baseline)^c^, median ± IQR	3 (0–5)	3 (0–5)	3 (0–5)	0.783

^a^t test for between-group comparison, ^b^chi-square for between-group comparison, ^c^Wilcoxon rank sum test for between-group comparison

*IQR* interquartile range, *Katz ADL* Katz index of independence in activities of daily living

### Effect on adverse outcomes

The independent variable of age that showed a potential difference between the intervention and control groups (Table 1) was inserted into the multivariate logistic regression model. Overall, the results showed that the control group was more likely to develop noninfectious complications (adjusted odds ratio: 3.01, *p* = 0.008) and a decline in ADLs (adjusted odds ratio: 11.20, *p* < 0.001). Other adverse outcomes, CAUTI and new nursing home admissions, did not show any significant difference between groups (Table 2).

**Table 2:** Logistic regressions of adverse outcomes between the intervention and control groups

Outcome measures	Unadjusted OR	*p*	Adjusted OR	*p*
	(95% CI)		(95% CI)	
CAUTI	1.82 (0.61–5.45)	0.281	1.58 (0.51–4.84)	0.422
Noninfectious complications	3.14 (1.35–7.27)	0.008	3.01 (1.32–6.81)	0.008
ADL decline	14.18 (4.33–46.35)	< 0.001	11.20 (3.68–34.00)	< 0.001
New nursing home admission	1.27 (0.35–4.58)	0.716	1.42 (0.35–5.68)	0.616

Reference: intervention group

*OR* odds ratio, *CI* confidence interval, *CAUTI* catheter-associated urinary tract infections, *ADL* activities of daily living

During the hospital stay, 41 of the 106 patients (38.6%) reported noninfectious complications due to indwelling urethral catheters. We observed that 14 patients (34.1%) developed noninfectious complications in the intervention group and 27 patients (65.8%) did so in the control group. Table 3 shows the percentage of reported noninfectious complications during the hospital stay after the urethral catheter was inserted. The most frequently cited noninfectious complications were blood in the urine (29.2%), protective restrictions (29.2%), and skin trauma related to catheter securement or catheter insertion (17.0%).

**Table 3:** Noninfectious complications associated with urethral catheter use

Noninfectious complications	Overall	Intervention group	Control group	*p*
	(*n* = 41)	(*n* = 14)	(*n* = 27)	
Blood in urine	12 (29.2%)	6 (42.8%)	6 (22.2%)	0.168
Protective constraint from accidental catheter removal	12 (29.2%)	0 (0.0%)	12 (44.4%)	0.003^a^
Trauma to skin related to catheter securement or catheter insertion	7 (17.0%)	4 (28.5%)	3 (11.1%)	0.205^a^
Pain or discomfort	6 (15.0%)	3 (21.4%)	3 (11.1%)	0.393^a^
Leakage or incontinence	2 (4.8%)	0 (0.0%)	2 (7.4%)	0.539^a^
Accidental removal	2 (4.8%)	1 (7.1%)	1 (3.8%)	1.000^a^

N (%)

*ADL* activities of daily living

^a^Fisher’s exact test

## Discussion

Here, we developed a novel intervention and explored the effect of that intervention to reduce noninfectious complications associated with indwelling urethral catheters in hospitalized older patients. The findings suggest that this novel intervention might effectively reduce noninfectious complications by 69% and ADL decline by 93.6% when used in addition to usual care in the acute setting.

With an increasing number of studies focusing on promptly removing urethral catheters that are no longer needed, two main types of removal strategies have been reported: urethral catheter removal reminders and urethral catheter removal stop orders [7]. The 14 studies reported the effectiveness of two types of removal strategies, and CAUTIs rates were reduced statistically in 6 of them; however, the outcomes were only identified in the ICU [21]. In line with this study, implementation of a novel intervention did not reduce the CAUTI rate in adult wards.

As no study to date has applied the intervention as we did to focus on the outcome of reducing noninfectious complications associated with indwelling urethral catheters in hospitalized older patients, we cannot make a comparison regarding this effect of this novel intervention with that in any previous study. Saint et al. [4] revealed that 27.4% of patients with catheters in place reported blood in their urine, and that result was similar to our finding (29.2%). Moreover, the prevalence of constraints used in hospitals ranged between 0 and 100% [22, 23]. Substantial differences in the prevalence of constraint use might depend on the type of ward, organizational policies, and the definition of constraint. Patient safety, including the prevention of accidental catheter removal, was the most frequently reported reason that all the patients in this study used a restraint glove as a protective constraint. These findings are consistent with previous studies [24–27]. Of note, the rate of constraint use to protect against accidental catheter removal in the intervention group was significantly lower than that in the control group. It is possible that more attention was given to the catheter in the intervention group; however, no such explanation was documented in the medical record.

The indwelling urethral catheter has even been referred to as a “one-point restraint,” which underscores the importance of limiting catheter use [28]. Saint et al. [4] showed that more than one-third of patients (39.5%) with catheters in place reported restrictions in ADLs. This result was similar to our finding in the control group (44.4%). Novel interventions might help patients not only limit the use of catheters but also provide a chance to increase their physical activity during hospitalization. Further study is needed to distinguish whether the effect of reducing ADL decline is caused by limiting catheter use or by increasing physical activity.

Some limitations of this study should be noted. First, the attrition rate was 11.3% of patients in the intervention group, which may result in some bias when interpreting the results. However, there were no differences in demographic and present health conditions in patients who were lost to the study in comparison to those who completed the follow-up period. Second, CAUTIs was based on the presence of the diagnosis in medical records, and not every patient in the study underwent urinary cultures after catheterization; therefore, the incidence of CAUTIs might be underestimated. Third, the different period of recruitment in intervention and control groups may cause the difference in patients’ characteristics. However, Table 1 showed no significantly difference between the groups in demographic factors (except age) and present health conditions. Fourth, compared to usual care, the additional time needed for nurses to integrate this novel intervention into daily practice may increase nurses’ workload. Further analysis on cost-effectiveness is needed to understand the value of novel interventions in the clinical setting. Finally, this study excluded patients who were undergoing surgery because the perioperative indications of appropriate use of urethral catheters varied by procedure. This limits these study results from being generalized to all hospitalized older patients.

## Conclusions

A novel intervention can be effective as a means of reducing noninfectious complications associated with indwelling urethral catheters in hospitalized older patients. This consensus intervention will help to standardize urethral catheter care in hospitalized older patients and assist in clinical decision-making for all health care professionals. Based on reducing inappropriate catheter use and promoting recovery of self-voiding function from the individual to the system, the results of this study provide information for clinical practice to reduce damage caused by urethral catheters in hospitalized older patients. More studies are needed to modify the current intervention to generalize it for all hospitalized older patients in clinical practice.

## Supplementary Information

**Additional file 1.**

## Data Availability

All relevant data are within the paper and its Supporting Information files.

## Abbreviations

CAUTIs: Catheter-associated urinary tract infection
ADL: Activities of daily living
CCI: Charlson comorbidity index
SPMSQ: Short portable mental status questionnaire
GDS-SF: Geriatric depression scale short-form
ICU: Intensive care unit
